# The use of Oral Health Impact on Daily Living (OHIDL) transition scale in measuring the change in oral health-related quality of life among older adults

**DOI:** 10.1186/s12903-021-01593-1

**Published:** 2021-05-03

**Authors:** Jian Liu, May Chun Mei Wong, Edward Chin Man Lo

**Affiliations:** 1 Department of Preventive Dentistry, Peking University School and Hospital of Stomatology, Beijing, China; 2 Dental Public Health, Faculty of Dentistry, The University of Hong Kong, Pokfulam, Hong Kong SAR China

**Keywords:** Longitudinal study, Oral health-related quality of life, Older adults, Psychometrics

## Abstract

**Background:**

This longitudinal study aimed to evaluate the longitudinal validity and reliability of the Oral Health Impact on Daily Living (OHIDL) transition scale and measure the perceived change in oral health-related quality of life (OHRQoL) after dental treatments among older adults.

**Methods:**

OHIDL was administered to older adults who sought dental treatments. Participants were asked to assess changes in impact for each OHIDL item retrospectively compared with that before the treatment. The responsiveness, minimal clinically important difference (MCID), internal consistency and test–retest reliability of the OHIDL transition scale were evaluated. Multiple linear regression was employed to predict the change in oral health impacts after dental treatment. Beta coefficients (β) and 95% confidence intervals (CI) were reported.

**Results:**

One hundred and seventy-six participants were followed-up with upon completing their dental treatments. The follow-up rate was 70.4% (176/250). The OHIDL transition score strongly correlated with the global rating of change (r_s_ = 0.76, P < 0.01). MCID was determined by participants who reported “a little improved” in the perceived oral health impacts, and their mean transition score was 3.3. Cronbach’s alpha of the transition scale was 0.87, and many items had a test–retest correlation of at least 0.60. Patients who perceived more oral health impacts at baseline as measured by the total intensity score (β = 0.32, 95% CI: 0.20, 0.44, P < 0.001) and those who had received endodontic treatment (β = 8.04, 95% CI: 4.36, 11.71, P < 0.001) would have more improvement in perceived oral health impacts.

**Conclusions:**

The OHIDL transition scale has good psychometric properties and is sensitive to change over time. After receiving dental treatment, most of the study’s older adults perceived a lower intensity of OHIDL.

**Clinical relevance:**

The OHIDL transition scale is a valid and reliable instrument to measure the change in OHRQoL after dental treatments.

## Background

With the shifting from a medical model to a social model in the health care system, the traditional biomedical end-points of clinical studies have been extended to include patient-centered measurements, such as quality of life (QoL) [1, 2]. According to Inglehart and Bagramian [3], oral health-related quality of life (OHRQoL) is assessed when the factors are centered on oral-facial concerns. Similar to the overall QoL, OHRQoL is multidimensional and encompasses different domains. The link between clinical variables, functional status, OHRQoL, and overall QoL is illustrated in the theoretical model developed by Sischo and Broder [4]. OHRQoL measurements have been adopted in large-scale epidemiological surveys in many countries to examine the trend in oral health and population-based needs assessment [4]. With the joint use of clinical indicators, OHRQoL measures have also been assessed to monitor the side effects of treatments and evaluate interventions' effectiveness in clinical trials from the patients’ perspective [5].

When detecting a change in OHRQoL, the most prevalent method is through calculating the change score, which is derived from subtracting the baseline score from the follow-up score after the intervention. Although using the change score seems appealing because of its simplicity and convenience, it has been criticized as a problematic and controversial approach. The criticisms mainly focus on its clinical meaning, its responsiveness that could be diminished by the ceiling/floor effect and its statistical properties [6].

Based on the change score, a statistically significant change in OHRQoL measurement does not necessarily mean a clinically significant difference that reflects the treatment effects, as the former can be influenced by the study sample size [7]. As a result, the concept of minimal clinically important difference (MCID) was proposed to attach clinical meaning to the change score [8] as assessed by the distribution-based methods (for example, standardized mean difference and effect sizes) and anchor-based methods. The distribution-based methods are limited in that they do not reflect the patients’ perspective of change in OHRQoL [9].

Individuals with the extreme measurement value at baseline, as denoted by the ceiling/floor effect, would not have space to show improvement or deterioration by calculating the change score. Consequently, it would fail to capture changes that happen at the follow-up evaluation [10, 11]. The ceiling/floor effect phenomenon has been widely reported in many longitudinal studies [12–15] and has also been found in dental research [16–20]. Besides being clinically meaningless and non-responsive, the change score may have low reliability [21–23] and regression to the mean [24]. Individuals with the lowest scores at baseline usually get the greatest improvement over time, while those with the highest scores tend to deteriorate.

Apart from calculating a change score, an alternative approach to measuring the change in OHRQoL is to ask the study participants to rate the perceived change retrospectively. This method has been applied for both a single-item global question and a multi-item scale, as indicated by the global transition judgments and transition scale, respectively [25]. The single-item global transition judgment has commonly been used as an external anchor to assess the overall change in OHRQoL [26–28]. Patients are asked to rate their change in OHRQoL over a specific time. Furthermore, using the global transition judgment to assess change also avoids the ceiling/floor effect. Despite the advantages, the global transition judgment has been challenged for its lack of sensitivity in detecting minor changes in OHRQoL that are important to people.

The transition scale is a multi-item scale consisting of a series of global transition judgments on different aspects of quality of life [25]. It shares the same advantages as the global transition judgment method. As a multi-item scale, it is more reliable than the single-item global transition judgment and more likely to reveal a change in OHRQoL in different dimensions [29]. The medical research field has applied it to assess the change in health-related quality of life. It has been shown to be reliable and sensitive to change and can enhance the instrument’s evaluative properties [29, 30]. Some studies have used the transition scale to assess change in OHRQoL [31–33]. However, only one instrument has been validated so far, the post-OHIP, which consists of 14 items in the original English version of OHIP-14 [32] recorded into three categories: “better”, “equal” and “worse” [33].

The Oral Health Impacts on Daily Living (OHIDL, 16 items in 7 domains) questionnaire was developed by the authors to measure the OHRQoL among Chinese older adults and its change after having received dental treatment [34]. A qualitative study was conducted to interview Hong Kong older adults about the life aspects that have been affected by their oral health problems. Also, what they expected (for those seeking treatment) or perceived (for those who have already received the treatment) the change in impacts after the dental treatment. A preliminary questionnaire of 21 items was developed from the semi-structured interviews using a framework approach [34]. Only a few participants mentioned perceived impacts in the social and psychological aspects due to the oral health problems they experienced. Chinese older adults have been found to have a greater level of acceptance in tooth loss, appearance and other oral health problems, and this is probably related to traditional Chinese health beliefs and culture [35–37]. Although social and psychological domains are valued components of both quality of life and health status, Chinese older adults are more likely to accept their oral health problems. They usually report a lower level of social impacts than older adults from Western cultures [38]. As a result, compared with the existing widely used instruments, even though OHIDL includes similar items to those in the Chinese version of OHIP-14 [39], GOHAI [40], OHRQoL-UK(W) [41] and OIDP [42], it contains fewer items related to social activities, psychological aspects and handicaps, i.e., embarrassment, confidence, work, romance, relaxing, miserable, unable to function, and unable to work. The constructed OHIDL has been further refined and its validity and reliability have been verified in a cross-sectional study, and using the intensity measurement to measure OHRQoL is recommended [35].

A transition scale of the OHIDL has also been proposed. This study aimed to evaluate the OHIDL transition scale’s validity and reliability among older adults in Hong Kong and measure the change in perceived OHRQoL after receiving dental treatments.

## Methods

### Participant recruitment

Dental clinics run by Non-Governmental Organizations (NGOs) in Hong Kong were approached for participant recruitment. Four dental clinics located in Kowloon and the New Territories areas agreed to participate in the study. At baseline, Chinese older adults aged 55 years and above who first attended the selected dental clinics seeking dental treatments were invited to participate in the study. In Hong Kong, a person aged 60 years or above is commonly considered as an older person. In this study, we have extended the age to a few years younger to facilitate the recruitment of participants. People with cognitive disorders, serious systemic diseases and communication difficulties, e.g., non-Cantonese speakers, were excluded. New patients were invited consecutively from April to October 2012. Patients who received any dental treatments were eligible for follow-up data collection. Older adults who only received dental examinations without any dental treatment were excluded from the follow-up evaluation. The study protocol was approved by the University of Hong Kong/Hospital Authority Hong Kong West Cluster Institutional Review Board (Reference no. UW12-081). The study’s purpose was explained to the participants using an information sheet, and a signed written consent form was collected from each participant.

The study sample size was determined by the number of items in the instrument and the desired estimate precision of the planned α coefficient [43]. The initial constructed OHIDL contained 21 items and with an expected α coefficient of 0.9 and 95% CI: 0.88–0.92, the required sample size was 204. It was estimated that 20% of the participants would drop out of the study and 30% would not receive any dental treatment after the first dental check-up. In order to follow up the required minimum number of participants to observe their changes in oral health impacts after having received dental treatments, the number of participants recruited at the baseline was increased to 306.

### Data collection

At baseline, before the participants received their dental treatments, they completed the OHDIL questionnaire interview. They were asked to indicate their oral health problems and symptoms, and these problems’ intensity level of impacts on daily living (16 items in 7 domains: Eating, Speaking, Appearance, Social, Psychological, Health and Finance, responses for each item ranged from “none” (0), “mild” (1), “moderate” (2), “severe” (3) to “very severe” (4)) [35]. The OHIDL intensity score was computed by summing up the responses for all 16 items. Information about each participant’s socio-demographic background, including age, gender, education level, people living with the participant, and purpose of the dental visit, were also collected. Within three months, and upon completing the dental treatments, the participants were invited to have a follow-up interview at their homes. They reported on the dental treatments they received. They also assessed the oral health problems and symptoms they were still experiencing, and the intensity level of oral health impacts, as in the baseline interview. The OHIDL change score was computed as the difference between the OHIDL intensity score at baseline and follow-up.

The OHIDL transition scale was designed to evaluate the effect of the received dental treatments in changing the oral health impacts on daily living. Following post-OHIP [33], participants were asked to rate the perceived changes in each impact in OHIDL after having received treatment compared to that before treatment. Instead of recording the changes in three categories only (“better”, “equal” and “worse”) as in post-OHIP. The response set for the changes in the OHIDL transition scale ranged from “very much improved” to “no change” to “very much deteriorated” and was recorded by a 7-point Likert scale from “+3” to “0” to “−3” correspondingly. The OHIDL transition score was computed by summing the responses for all 16 items.

Two global questions were asked in the follow-up interview: (1) the overall perceived change in oral health impacts due to the treatments (from “very much improved” to “no change” to “very much deteriorated”), and (2) the satisfaction with the treatments received (from “very dissatisfied” to “very satisfied”). To avoid bias, the interviewer was blind to the baseline information when interviewing the follow-up participants.

### Statistical analysis

The OHIDL transition and change scores were computed. The OHIDL transition scale’s longitudinal (construct) validity was evaluated through its responsiveness and MCID. Responsiveness of the transition score was assessed by examining the relationships among the global rating of change, the transition score, and the change score using the Spearman rank correlation. Variations of the transition score across the different categories of the global rating of change were also compared with the change score. OHIDL transition score was expected to have a stronger correlation with the global rating when compared to the OHIDL change score. The transition score’s MCID and that of the change score were determined with the anchor-based method. The mean score for participants who reported “a little improved” and “a little deteriorated” in the global rating of change was considered MCID. OHIDL transition score was expected to have a smaller MCID than that of OHIDL change score.

The reliability was evaluated through internal consistency and test–retest reliability. Internal consistency of the transition scale was tested using Cronbach’s alpha coefficient. A value of 0.7 was regarded as an acceptable level of internal consistency. Test–retest reliability was only assessed at the follow-up interview. For the last 80 participants who attended the follow-up interviews, five items on the OHIDL (25%, excluding the additional items) were randomly selected to be re-administered with the transition measurements. The duplication was carried out right after the follow-up interview. In total, 10% of the items were duplicated. The correlation coefficient between the same item's two responses was calculated to assess the test–retest reliability for the measurements.

The association between the change in oral health impacts and the satisfaction with treatment was explored through Kruskal–Wallis one-way ANOVA. Using the Mann–Whitney U test, changes in oral health impacts were compared between the participants who had received different dental treatments. Treatments that were less commonly received (complete denture, fixed prosthesis, denture repair, crown, implant and root planning) were grouped as other dental treatments. Multiple linear regression was employed to predict the change in oral health impacts after having received dental treatment. The transition score was the dependent variable. Age, gender, education level, people living together, the purpose of the dental visit, baseline intensity score, number of oral health problems before receiving treatments and the treatments the participants had received were the independent variables. Beta coefficient (β) and 95% confidence intervals (CI) were calculated. Variable selection was carried out through the forward method. Factors with a P-value less than 0.05 were selected to enter into the model. Data were analyzed using SPSS version 20. The level of statistical significance for all tests was set at 5%.

## Results

Among the 306 older adults who participated in the baseline data collection, 56 were excluded from the follow-up evaluation because they did not receive any dental treatment. Seventy-four participants refused to be interviewed again after receiving dental treatments; some said the interview process was cumbersome, and the others did not specify a reason. In total, 176 participants were re-interviewed upon completing their dental treatments (mean age = 60.1 years, SD = 8.1 years). The follow-up rate was 70.4% (176/250). There was no statistically significant difference detected between the participants' socio-demographic background at follow-up and those who participated in the baseline interview (Table 1). Most of the participants were problem-driven in terms of their motivation for attending dental clinics.

**Table 1:** Characteristics of participants at the follow-up evaluation and those who dropped out

Socio-demographic factors	Follow up n = 176 (%)	Drop out n = 130 (%)	P*
*Age*			0.629
55–59	18 (10.2)	10 (7.7)	
60–64	44 (25.0)	28 (21.5)	
65–69	38 (21.6)	23 (17.7)	
70–74	29 (16.5)	28 (21.5)	
75–79	27 (15.3)	21 (16.2)	
≥ 80	20 (11.4)	20 (15.4)	
*Gender*			0.407
Male	76 (43.2)	50 (38.5)	
Female	100 (56.8)	80 (61.5)	
*Education*			0.485
No formal education	25 (14.2)	25 (19.2)	
Primary school	75 (42.6)	47 (36.2)	
Secondary school	55 (31.3)	45 (34.6)	
Tertiary education or above	21 (11.9)	13 (10.0)	
*People living with*			0.333
Single	34 (19.3)	18 (13.8)	
Spouse or other older adults	59 (33.5)	52 (40.0)	
Children or other younger people	83 (47.2)	60 (46.2)	
*Purpose of dental visit*			0.106
Regular dental check up	37 (21.0)	18 (13.8)	
Problem driven	139 (79.0)	112 (86.2)	

*Chi-square test

At baseline, the participants reported 4.7 oral health problems on average (SD = 2.3), ranging from 0 to 11. Food catching was the most prevalent (77.3%), followed by missing teeth (51.7%), tooth sensitivity (46.0%) and mobile teeth (46.0%) (Table 2). A substantial proportion of participants reported pain-related symptoms. After the dental treatments, the mean number of oral health problems experienced significantly decreased to 2.7 (paired t-test, P < 0.05). The prevalence of self-reported oral health problems was also reduced. Prevalence of mobile teeth showed the greatest decrease (from 46.0 to 15.9%), followed by toothache (from 40.3 to 10.8%), caries (from 31.3 to 11.4%) and gum pain (from 31.3 to 13.1%). However, food catching had little change (from 77.3 to 72.7%).

**Table 2:** Prevalence of self-reported oral health problems before and after dental treatment (n = 176)

Oral health problem	Prevalence (%)	
	Before treatment	After treatment
Food catching	77.3	72.7
Missing teeth	51.7	40.3
Mobile teeth	46.0	15.9
Tooth sensitivity	46.0	28.4
Toothache	40.3	10.8
Bad breath	38.6	26.1
Gum pain	31.3	13.1
Dental caries	31.3	11.4
Gum bleeding	34.7	16.5
Calculus	33.5	15.3
Unfit denture	11.4	10.2
Sore jaw	7.4	4.0
Others	27.8	8.5

Among the 176 study participants, simple treatments were the most commonly received dental treatments and usually required only a single dental visit, such as for scaling (54%), filling (35.8%) and extraction (34.7%). About one-fifth of the participants received removable partial denture (22.2%), while nearly one-tenth received root canal treatment (9.1%) and medication (9.1%). Meanwhile, the more complex dental treatments such as complete denture (7.4%), crown (5.1), root planing (2.3%), bridge (1.1%), denture repair (1.1%) and implant (0.6%) were less commonly received. Eighty-one (46.0%) participants had received only one treatment item.

### Longitudinal validity and reliability of the OHIDL transition scale

The participants were asked to rate the intensity level of the oral health impacts listed in the OHIDL before and after the treatments, and the perceived change in these oral health impacts compared to those experienced before the dental treatment (the transition scale). For individual oral health impact items, over 50% of the older adults reported no change at the follow-up interview (Table 3). Items in the “Social” domain showed the smallest change, with 86.4% (“uncomfortable to eat in front of people”) and 87.5% (“self-conscious”) of the participants perceived no change. Less than 5% of the participants reported “moderately” to “very much deteriorated” for any perceived oral health impacts. The most prevalent negative change observed was “financial burden”, with around 16.0% of the participants perceiving a little deterioration or more. Items included in the “Eating” domain showed relatively more improvement than the other domains after dental treatments, ranging from 11.3% (“meal interruption”) to 38.1% (“eating discomfort”) of the participants reporting positive changes with only 1.1% to 8.5% showing “very much improved”.

**Table 3:** Frequency (percent in parenthesis) distribution of the responses to each item in the OHIDL transition scale

Item	OHIDL transition scale n = 176 (%)						
	Very much deteriorate (score = − 3)	Moderate deteriorate (score = − 2)	A little deteriorate (score = − 1)	No change (score = 0)	A little improved (score = 1)	Moderate improved (score = 2)	Very much improved (score = 3)
*Eating*							
Food limitation	0 (0)	0 (0)	10 (5.7)	119 (67.6)	19 (10.8)	16 (9.1)	12 (6.8)
Eating discomfort	0 (0)	5 (2.8)	8 (4.5)	96 (54.5)	23 (13.1)	29 (16.5)	15 (8.5)
Chewing difficulty	0 (0)	2 (1.1)	8 (4.5)	107 (60.8)	25 (14.2)	22 (12.5)	12 (6.8)
Eating time prolonged	0 (0)	2 (1.1)	9 (5.1)	126 (71.6)	18 (10.2)	16 (9.1)	5 (2.8)
Meal interruption	0 (0)	1 (0.6)	6 (3.4)	149 (84.7)	9 (5.1)	9 (5.1)	2 (1.1)
Less flavor in food	0 (0)	1 (0.6)	7 (4.0)	141 (80.1)	12 (6.8)	8 (4.5)	7 (4.0)
*Speaking*							
Speaking difficulty	1 (0.6)	2 (1.1)	6 (3.4)	144 (81.8)	16 (9.1)	1 (0.6)	6 (3.4)
*Appearance*							
Appearance affected	0 (0)	1 (0.6)	4 (2.3)	129 (73.3)	21 (11.9)	11 (6.3)	10 (5.7)
Avoiding smile	0 (0)	0 (0)	3 (1.7)	146 (83)	9 (5.1)	8 (4.5)	10 (5.7)
*Social*							
Uncomfortable to eat in front of people	0 (0)	0 (0)	3 (1.7)	152 (86.4)	5 (2.8)	8 (4.5)	8 (4.5)
Self-conscious	0 (0)	0 (0)	2 (1.1)	154 (87.5)	5 (2.8)	7 (4.0)	8 (4.5)
*Psychological*							
Worried	0 (0)	1 (0.6)	7 (4.0)	120 (68.2)	25 (14.2)	15 (8.5)	8 (4.5)
Mood affected	0 (0)	0 (0)	9 (5.1)	124 (70.5)	20 (11.4)	13 (7.4)	10 (5.7)
*Health*							
Headache	0 (0)	0 (0)	2 (1.1)	148 (84.1)	5 (2.8)	10 (5.7)	11 (6.3)
Sleep interrupted	0 (0)	0 (0)	2 (1.1)	145 (82.4)	7 (4.0)	13 (7.4)	9 (5.1)
*Finance*							
Financial burden	1 (0.6)	8 (4.5)	20 (11.4)	141 (80.1)	3 (1.7)	1 (0.6)	2 (1.1)

The OHIDL transition and change scores were computed and correlated with the global rating of change to evaluate the transition scale's responsiveness. The transition score had a higher correlation with the global rating of change (r_s_ = 0.76, P < 0.001) than that of the change score and global rating of change (r_s_ = 0.37, P < 0.001). The transition score varied in the expected direction, and the results consistently corresponded to the global rating of change categories (Table 4). However, the change score showed inconsistent results with the global rating when the participants reported no overall change or a little deterioration in oral health impacts. Besides, compared to the change score, the transition score also demonstrated a better discriminating property with more diversity among the different global rating categories. The OHIDL transition scale demonstrated better responsiveness than the change score. MCID was determined by the participants with “a little improved” in the perceived oral health impacts. Participants with “a little deteriorate” in oral health impact were not used to determine MCID because of the limited sample size (n = 9). For the mean transition score, MCID was 3.3, which was smaller than that of the change score (4.3). These results show that the transition scale demonstrated a good longitudinal validity.

**Table 4:** Transition score and change score according to the global rating of change (mean, 95% confidence interval)

Global rating of change	n	Transition score		Change score	
		Mean	(95% CI)	Mean	(95% CI)
Much deteriorated	0	–	–	–	–
Moderately deteriorated	2	− 5.5	(− 75.4, 64.4)	2.0	(2.0, 2.0)
A little deteriorated	9	− 3.4	(− 5.8, − 1.2)	0.1	(− 6.8, 7.0)
No change	63	0.4	(< − 0.1, 0.9)	2.1	(1.0, 3.2)
A little improved*	43	3.3	(1.6, 5.0)	4.3	(2.4, 6.1)
Moderately improved	47	9.9	(8.1, 11.7)	7.2	(4.7, 9.7)
Very much improved	12	20.7	(13.3, 28.1)	16.4	(9.0, 23.8)
Total	176	4.8	(3.6, 6.0)	4.7	(3.7, 6.0)
Spearman rank correlation		0.76		0.37	

*Used to determined minimal clinically important difference (MCID)

Regarding reliability, Cronbach’s alpha of the transition scale was 0.87, indicating good internal consistency. The test–retest reliability for individual items mostly ranged from 0.61 (“financial burden”) to 1.00 (“headache”), except for “eating time prolonged” (0.28), “speaking difficulty” (0.50) and “less flavor in food” (0.57). The transition scale demonstrated satisfactory reliability.

### Satisfaction and change in perceived OHRQoL after dental treatment

After the dental treatment, over half (58.0%) of the older adults rated their overall perceived oral health impacts as improved. However, 35.8% of the older adults reported their oral health impacts as no change and 6.2% reported deteriorated. Close to two-thirds (63.1%) of the participants were satisfied or very satisfied with the treatment they received, while 29.0% were neither satisfied nor dissatisfied. The more satisfied patients with the received treatments had higher mean OHIDL transition scores (satisfied/very satisfied: 6.6; neither satisfied nor dissatisfied: 2.1; dissatisfied/very dissatisfied: 0.1, Kruskal–Wallis 1-way ANOVA, P < 0.001).

Table 5 shows the association between the dental treatment received and the transition score. The older adults who received root canal treatment (12.8 vs 4.0, Mann–Whitney U test, P < 0.001) had a significantly higher mean transition score for the individual treatments. Also, older adults who received other treatments (complete denture, fixed partial denture, denture repair, crown, implant and root planning) had a significantly higher mean OHIDL transition score (8.0 vs. 4.2, Mann–Whitney U test, P = 0.041).

**Table 5:** Mean transition score for participants receiving different dental treatments

Dental treatment		Mean transition score	P**
Scaling	Yes (n = 95) No (n = 81)	4.1 5.6	0.075
Filling	Yes (n = 63) No (n = 113)	4.6 4.9	0.397
Extraction	Yes (n = 61) No (n = 115)	5.5 4.4	0.249
Medication	Yes (n = 16) No (n = 160)	7.4 4.5	0.204
Removable partial denture	Yes (n = 39) No (n = 137)	7.0 4.2	0.078
Root canal treatment	Yes (n = 16) No (n = 160)	12.8 4.0	< 0.001
Other dental treatments*	Yes (n = 29) No (n = 147)	8.0 4.2	0.041

*Complete denture, fixed prosthesis, denture repair, crown, implant and root planing were grouped as other dental treatments

**Mann–Whitney test

Multiple linear regression analysis was carried out to investigate the effect of various factors on the OHIDL transition score. In the final model, 23% of the variations in the OHIDL transition scores could be explained. Patients with a higher baseline OHILD intensity score (β = 0.32, P < 0.001) and those who had received a root canal treatment (β = 8.04, P < 0.001) would have more improvement in perceived oral health impacts (Table 6).

**Table 6:** Final model to predict change in OHIDL after dental treatment

Transition in OHIDL	β	95% CI	P	R^2^
Baseline total intensity score	0.32	0.20, 0.44	< 0.001	0.23
Root canal treatment	8.04	4.36, 11.71	< 0.001	

## Discussion

This study evaluates the OHIDL transition scale’s longitudinal validity and reliability. The results show that the transition scale is sensitive to change over time and possesses good longitudinal validity and reliability. After receiving dental treatment, older adults perceived fewer oral health problems and positive changes in oral health impacts on daily living.

Considering the subjectivity of quality of life assessment, people may refer to various internal reference systems when they answer the same question. Because individuals’ circumstances may change with time, the basis on which the individuals make a QoL judgment may also change [44]. Response shift refers to a change in the meaning of one's evaluation of a construct as a result of a change in one's internal standards of measurement, a change in one's values, or a change in one's definition of the construct [45]. Individuals who are coping with an illness may value health states differently throughout the course of the disease or treatment. QoL measures currently used in clinical research are not designed to account for response shifts but assuming that people would respond consistently on the measurement scales and that scales are directly comparable across individuals and over time [46]. Considering the response shift, assessing change in OHRQoL with prospective measurements, such as change score, may cause biases to estimate the treatment effect. As a result, conventional prospective assessments of change based on self-reports may overestimate or underestimate the intervention or the effect of illness [47, 48].

This study tried to evaluate the change in OHRQoL through a transition scale retrospectively. When people talk about a situation to be “better” or “worse”, the meaning may also differ from person to person. Being better is not only reflected in changes in the state of the disorder (resolution) but could be an adjustment of life to work around the disorder (readjustment) or an adaptation to living with the disorder (redefinition) [49]. Thus, people may respond differently over time, not only because their quality of life has changed due to disorder or treatment but also because they may have changed their views on what quality of life means to them. This consideration is important for assessing treatment outcomes, as changes in quality of life may reflect response shift, treatment effects, or a complex combination of the two. When the transition scale is used as the outcome measurement to retrospectively evaluate the change in OHRQoL as an evaluation of the effectiveness of a certain treatment, it is necessary to interpret the results with caution and not to exaggerate or underestimate the treatment effect. Study design with control groups and incorporating the objective clinical indicators would be needed [50]. Besides, some researchers suggest adding qualitative questions following the transition questions, such as asking "why do you report being <better, worse or about the same>?" to further explore the reasons for answering and identify the root causes of the changes [51].

In this study, the global rating of change in perceived oral health impacts was used to compare the transition scale's longitudinal validity and the change score. It was considered an indicator and served as an anchor for the overall change in OHRQoL. Compared to the change score, the transition scale had better agreement with the global rating’s change categories, as higher positive correlations with the global rating were found. However, the relationships with global rating were not that obvious for the change score. The transition scale also had larger diversity among different global rating categories, indicating higher sensitivity than the change score in terms of detecting change.

When the OHIDL change score is zero, there is no change in OHRQoL or the change cannot be reflected due to the ceiling/floor effect. In this study, 13% of the participants reported having perceived no oral health impacts on their daily lives at baseline, and 31% of the participants reported low oral health impacts with an intensity score of less than 5. For participants with low impact at baseline, the OHIDL transition score showed positive change after treatment, while the change score detected no difference. After treatment, deterioration in oral health impacts was detected by using the change score for the participants who reported no impact before dental treatment. This observation may be explained by the ceiling/floor effect, which is viewed as the change score’s methodological flaw. The improvement cannot be captured by the change score when individuals already reported the lowest possible value of impacts at the baseline. In this situation, OHRQoL is only “allowed” to remain stable or deteriorate over time. In contrast, employing the transition score allows an individual to report improvement after receiving dental treatments regardless of the status of the perceived impact before treatment [10].

Interpreting the change data would be more confident with the transition scale as it includes the individual’s subjective valuation. It would solve the difficulty of what degree of change is necessary to be considered meaningful. Post-OHIP has been developed to use the transition scale to assess change in OHRQoL and assess the effectiveness of prosthodontic treatment [32, 33]. Post-OHIP consisted of 14 items, and the responses were recorded into three categories: “better”, “equal” and “worse”. Compared with post-OHIP, the change in OHRQoL in the OHIDL transition scale was evaluated in a 7-point Likert scale in this study. Considerable variation was found among “a little improvement”, “moderate improvement” and “a great improvement” suggesting it is more sensitive in quantifying the magnitude of change.

The effectiveness of dental treatment can be measured through different indicators, e.g., reduction in oral health problems, improvement in OHRQoL or satisfaction with treatment. In this study, participants who reported fewer oral health problems at baseline had higher transition scores at the follow-up, indicating the dental treatments’ positive effects. However, after dental treatment, a high proportion of participants were still reporting food catching (72.7%) and missing teeth (40.3%), which was only slightly lower than those found at baseline, indicating potential treatment needs. Many of the low-income study participants received dental treatment funded by the Comprehensive Social Security Assistance (CSSA) provided by the Hong Kong SAR government. The treatment plans were restricted by the limited funding regardless of the treatment need. As a result, these older adults usually chose to receive simple dental treatments, such as scaling, filling and extraction that could be covered by the CSSA and to avoid paying the high costs of advanced dental treatments out of their own pockets. Alternatively, some participants may choose to leave these oral health problems unsolved, possibly because the problems were not severe enough to affect OHRQoL. The findings highlight dental neglect among Hong Kong older adults, which is widespread and associated with socio-demographic factors and OHRQoL [52]. People can live with chronic oral health problems without seeking any dental treatment. The dental neglect continues until the accumulative eating function impairment can no longer be coped with, i.e., the problems are severe enough to affect OHRQoL. A previous study found that adults in Hong Kong were more prepared for tooth loss than adults in the UK [53]. This may be because they have developed a set of strategies to cope with various life stresses and strains [54]. Another possible reason is that the emotional effects of tooth loss are not marked among older adults in Hong Kong [55]. The negative oral health impacts can be minimized through psychological adjustments, such as changes in expectations, lifestyles and living environment, and using dental devices [56].

Around two-thirds of the participants in this study were satisfied with the dental treatment they received. A significantly higher transition score was only observed among patients who felt very satisfied with the treatment received. The treatment satisfaction indicators and change in OHRQoL imply different measurement outcomes, which can dramatically change the conclusion of the treatment effectiveness. Patients may not be satisfied with the received treatment even if there is an improvement in OHRQoL, although these two factors tend to be significantly associated with one another [57]. Treatment satisfaction can also be influenced by many factors, e.g., the quality of health care, access, and treatment cost [58]. Despite the high level of treatment satisfaction, the perceived change in OHRQoL after dental treatment was low, with over 50% of the study participants reporting no change on each item. The low perceived oral health impacts at baseline may be one reason for this. Items in the “Social” domain showed the smallest change, consistent with previous studies that Chinese older adults reported a low level of social impacts [36–38]. It is not surprising to find the social domain showed the smallest change after the study participants received dental treatment. Another reason is that the common treatments the participants in this study received were relatively simple procedures, and only a few participants received prosthodontic or other advanced treatments. This finding is expected in Hong Kong older adults, mainly because of the expensive cost of comprehensive dental treatment.

Findings in this study highlight the effectiveness of endodontic treatment in improving the OHRQoL of older adults, and likely, the teeth that required endodontic treatment were heavily broken down and painful. The success of this treatment removed the pain and restored function, which had significantly improved the OHRQoL. Note that the treatments were not mutually exclusive, i.e., older adults who received advanced treatment may also have received some simple treatments. Thus, the more significant improvement as observed in these older adults may be attributed to the combined treatment effect. Since more than half of the participants had received multiple treatments, the interaction effect existed between individual treatment items. The co-existence of multiple treatments may confound each type of dental treatment’s effectiveness. However, limited by the sample size, it was not practical or feasible to explore all the combinations in this study. Future studies should be conducted to investigate the effectiveness of specific dental treatment in improving OHRQoL using the OHIDL in a controlled setting.

This study has several limitations. First, the study participants were recruited from four dental clinics run by NGO, it was a convenience sample and might not be representative. Second, the small number of study participants who had received complex dental treatment may affect the accuracy of the study results. A deliberate study design with a larger sample size and including older adults with more diverse socio-demographic backgrounds from a representative sample is demanded in future study. Third, this study was also limited by the repeated interviews for the test–retest reliability which were carried out right after the follow-up interview, instead of after some time, e.g., several days or a week later. Since the follow-up data were collected in individual home visits, it would be disturbing to approach the participants one more time. Although the test–retest information could also be collected through other ways, such as telephone interview, there could be a risk of bias because of the change in the mode of administration. Because of time constraints and being burdensome for the older adults to repeat answering all the items in the OHIDL transition scale, only five items were randomly selected (different items were selected in the different repeated interviews) for duplication during the follow-up evaluation. Although the test–retest reliability was evaluated for each item only instead of the whole scale, it is believed the result still supports the reliability of OHIDL. Future studies may consider carrying out the duplication with a longer time interval. However, caution needs to be taken when choosing the time interval because OHRQoL is a dynamic concept, and respondents’ perceptions can quickly change based on their expectations and experience [59].

## Conclusion

This study showed that the OHIDL transition scale has good psychometric properties and is sensitive to change over time. Using the transition scale in measuring a change in OHRQoL eases the interpretation of the change in OHRQoL after dental treatments and avoids the ceiling/floor effect. After receiving dental treatment, most older adults perceived fewer oral health problems and a lower intensity of oral health impacts on daily living. Older adults who had received advanced treatment tended to have more improvement than those who received simple treatments. Most of the study participants were satisfied with the dental treatment they received.

## Data Availability

Datasets analyzed during the current study are available from the corresponding author on reasonable request.

## Abbreviations

OHIDL: Oral Health Impact on Daily Living
OHRQoL: Oral health-related quality of life
MCID: Minimal clinically important difference
NGOs: Non-governmental organizations
QoL: Quality of life
